# Cardiometabolic Health and Bariatric Surgery: A 25-Year Longitudinal Cohort Study in CARDIA Participants

**DOI:** 10.1097/AS9.0000000000000609

**Published:** 2025-09-04

**Authors:** Brian T. Steffen, Sayeed Ikramuddin, David R. Jacobs, Kristi L. Kopacz, Daniel Duprez, Ankeet S. Bhatt, Jamal S. Rana, John J. Carr, Xia Zhou, Lyn M. Steffen

**Affiliations:** From the * Division of Computational Health Sciences, Department of Surgery, University of Minnesota School of Medicine, Minneapolis, MN; † Department of Surgery, University of Minnesota School of Medicine, Minneapolis, MN; ‡ Division of Epidemiology and Community Health, University of Minnesota School of Public Health, Minneapolis, MN; § University of Minnesota Physicians, Minneapolis, MN; ‖ Division of Research, Kaiser Permanente, Oakland Medical Center, Oakland, CA; ¶ Department of Radiology and Vanderbilt Translational and Clinical Cardiovascular Research Center (VTRACC), Vanderbilt University Medical Center, Nashville, TN.

**Keywords:** bariatric surgery, cardiometabolic risk factors, obesity

## Abstract

**Objective:**

Compare longitudinal cardiometabolic health outcomes among individuals who underwent bariatric surgery (BarS) with nonsurgical controls.

**Background:**

BarS is well-established for inducing profound weight loss and improving cardiometabolic health, but it remains unclear whether patients achieve long-term cardiometabolic health consistent with the attained lower weight status.

**Methods:**

Cohort study participants who underwent any BarS procedure between 1987 and 2021 (n = 94) were paired with sex- and body mass index (BMI)-matched nonsurgical controls (n = 282) at the nearest postoperative cohort exam visit (2.8 ± 1.7 years following surgery). A mixed model tested differences between BarS cases and nonsurgical matched controls, adjusting for age, sex, race, field center, and maximal education attainment. Intermediate cardiometabolic endpoints and incident diabetes and metabolic syndrome, were examined at follow-up exam visits.

**Results:**

Approximately 7.5 years following their procedures, those who underwent BarS showed higher BMIs than matched controls (+2.8 kg/m^2^); however, the BarS group showed significantly lower mean fasting levels of glucose (−6.5 mg/dL; *P* = 0.03), insulin (−2.75 μU/mL; *P* = 0.01), low density lipoprotein cholesterol (−20.0 mg/dL; *P* < 0.001), C-reactive protein (log-transformed) (−0.42; *P* = 0.002), homeostasis model assessment-estimated insulin resistance (−0.75; *P* = 0.02), and higher mean high density lipoprotein cholesterol (+11.4 mg/dL; *P* < 0.001) compared to matched controls. BarS cases showed lower incidence of diabetes (1.8% vs 11.7%; *P* = 0.007) and nominally lower MetS (13.7% vs 22.3%; *P* = 0.23).

**Conclusions:**

We found no evidence of lasting adverse cardiometabolic health consequences of severe obesity in a sample of cohort participants who underwent a BarS procedure. On average, BarS cases showed features of better cardiometabolic health than postoperative-matched nonsurgical controls who followed a more moderate trajectory of obesity.

## INTRODUCTION

Bariatric surgery (BarS) is the most effective tool for lasting weight loss in individuals with severe obesity—inducing 30% in total body weight or 10 kg/m^2^ body mass index (BMI) units, depending on age,^1^ sex,^2^ and patient health.^3^ Cardiometabolic improvements are also typical, including improved glucose control and lower fasting insulin, blood lipids, and blood pressure.^1,4–7^ To date, studies have consistently shown that individuals who undergo a BarS intervention show better cardiometabolic outcomes than preoperative-matched counterparts in both observational studies^8–11^ and controlled trials.^12–14^ These studies have demonstrated and confirmed the benefits of BarS compared to an individual at a similar weight status, but they are not designed to test whether BarS patients may achieve the cardiometabolic health of those at their postoperative weight or whether BarS may reverse the cardiometabolic consequences associated with years of severe obesity before the procedure.

Obesity and severe obesity promote, or are otherwise associated with, the pathogenesis of numerous chronic metabolic and cardiovascular diseases.^15–17^ Critically, duration and severity of obesity have been associated with greater risks of all-cause mortality and cardiometabolic disease^17,18^ as well as higher blood pressure, dyslipidemia, and a decline in glucose control.^19,20^ In the context of BarS, many patients have spent years, even decades, with obesity and severe obesity, and the consequences of this exposure over time may still be evident following surgical intervention.

No observational study has examined whether BarS reverses the accrued health consequences associated with obesity on cardiometabolic risk profiles or outcomes over a long-term follow-up period. This study compared the longitudinal cardiometabolic health of individuals who underwent a BarS procedure with sex-BMI-matched controls at the closest postoperative exam visit. Given the years of severe obesity or compromised metabolic health in the BarS group before surgery, we hypothesized that these individuals would show indications of worse cardiometabolic health over the 7.5-year postsurgical follow-up period compared to nonsurgical controls.

## MATERIALS AND METHODS

### Study Population

The Coronary Artery Risk Development in Young Adults (CARDIA) is a prospective cohort study that aims to examine factors that influence coronary heart disease development during young adulthood. CARDIA participants were 18 to 30 years old at baseline and split relatively evenly within each clinic on age, race, sex, and educational attainment at baseline. CARDIA clinic visits occurred at years 0, 2, 5, 7, 10, 15, 20, 25, 30, and 35. The variables examined here were measured at most or all visits. Over 70% of surviving participants attended each clinic visit except for Y35, which was conducted during the COVID-19 pandemic.

Bariatric surgeries (N = 113) occurred throughout the cohort study follow-up and were either identified by self-report in the medical history questionnaire (the calendar year of surgery was not always specified) or from a hospitalization report (hospitalization date was typically specified). Nineteen participants who indicated that they received BarS were excluded because they had sparse clinic attendance. The surgical technique used was not always known, but Roux-en-Y gastric bypass (n = 27) and vertical sleeve gastrectomy (n = 15) were the most common procedures among those who specified their procedure. Seven study participants reported a lap band.

### Laboratory Measurements

Fasting glucose and insulin were measured using respective hexokinase and radioimmunoassay assays at every exam visit except for years 2 and 5. Total cholesterol, triglycerides, and high-density lipoprotein cholesterol (HDL-C) were assessed using standardized assays, and low-density lipoprotein cholesterol (LDL-C) was calculated by the Friedewald equation. High-sensitivity C-reactive protein (hsCRP) was measured at years 7, 15, 20, and 25 using high-sensitivity nephelometry (BNII nephelometer, Dade Behring, Eschborn, Germany). The homeostasis model assessment-estimated insulin resistance (HOMA-IR) index was calculated using the standard formula: fasting insulin (μU/dL) × fasting glucose (mg/dL)/405.

### Blood Pressure and BMI

Resting blood pressure was measured in participants in a seated position 3 times. The means of the 2nd and 3rd systolic and diastolic readings served as the blood pressure outcomes. Height and weight were measured by a stadiometer and beam balance scale, respectively, and BMI was calculated as weight in kilograms divided height in meters squared (kg/m^2^).

### Metabolic Disease

The presence of diabetes or metabolic syndrome was evaluated at each exam visit. Diabetes was defined in accordance with American Diabetes Association criteria.^21^ Metabolic syndrome was defined by the presence of at least 3 of 5 factors: abdominal obesity (waist circumference >88 cm in women or >102 cm in men); high blood pressure (systolic blood pressure ≥130 mm Hg or diastolic blood pressure ≥85 mm Hg); hyperglycemia (fasting blood glucose ≥100 mg/dL); HDL-C <40 mg/dL in men or <50 mg/dL in women; triglyceride levels ≥150 mg/dL.

### Covariates

Questionnaires assessed demographic (age, sex, race, and education) and lifestyle characteristics. A separate questionnaire was administered to assess physical activity.^22^ Physical activity scores were calculated based on time spent in activities, weighted by estimated energy expenditures.

### Matching and Statistical Analysis

SAS, version 9.4 (SAS Institute, Cary, NC), was used to analyze the data. Of the 113 participants who reported a BarS procedure, 94 attended a sufficient number of exams for analysis. The 94 surgical cases were each matched on sex and BMI (within 3 kg/m^2^) at the clinic visit directly following surgery with 3 nonsurgical controls. Controls were selected randomly from within the set of participants meeting the BMI criteria. Controls were pooled for the analysis and data were treated as frequency-matched between BarS and controls.

Characteristics are presented for BarS cases and pooled nonsurgical controls. Unadjusted means and standard errors for each outcome over 8 CARDIA exams were computed and aligned according to scheduled exams for each participant so that T_0_ and T_1_ were the exams immediately preceding and following the surgery, respectively. Missing data, either because the surgery occurred in recent years, resulting in fewer follow-up visits or because of failure to attend a given visit, were assumed to be missing at random. Lower numbers of BarS cases were available at T_3_ (n = 44) and T_4_ (n = 31); however, at least 60 BarS cases were available for analysis between T_−3_ and T_2_ time points. A mixed model analysis (SAS PROC MIXED, using the sandwich estimator for standard error estimates) was conducted across T_0_, T_1_, and T_2_ for each outcome as the dependent variable (linked by case-control quadruplet, ordered as the outcome value for surgical case, control 1, control 2, and control 3) and regressed on surgical case-control status (thus pooling all controls). The within-examination surgical case – pooled control difference net difference (subtracting the T_0_ values) and its standard error for each outcome variable were estimated in this way. The significance threshold was fixed at *P* < 0.05; however, interpretive caution was exercised due to the number of outcomes tested.

Finally, a secondary analysis of bariatric surgical cases and nonsurgical controls was performed as above, but groups were matched at the preoperative time point T_0_ to confirm the results of previous studies. Due to extreme obesity at T_0_, BMI was matched to within 5–9 kg/m^2^ in 19 participants, and 9 participants had fewer than 3 matched controls.

## RESULTS

Demographic, lifestyle, and clinical characteristics of CARDIA participants who underwent a bariatric procedure and sex-, BMI-matched controls are shown in Table 1. Characteristics of the BarS group are specified for both the preoperative exam (T_0_, 2.3 ± 1.6 years before BarS) and postoperative exam (T_1_, 2.8 ± 1.7 years following BarS). The expected anthropometric and cardiometabolic improvements were evident in BarS cases between preoperative T_0_ and postoperative T_1_ time points. Unadjusted means and standard errors for each outcome over 8 CARDIA exams generally showed the higher presurgery risk levels in surgical cases and substantive postsurgical improvements (Figures S1–S13, https://links.lww.com/AOSO/A533).

**TABLE 1.** Demographic, Lifestyle, and Clinical Characteristics of CARDIA Participants

Characteristic	Surgery Cases (T_0_)*	N	Surgery Cases (T_1_)†	N	Matched Controls (T_1_)	N
Age (year), mean (SD)	43.6 (7.3)	94	49.8 (7.6)	81	50.4 (8.9)	273
Age at surgery, mean (SD)	46.5 (8.0)	94	46.5 (8.0)	94	N/A	–
Female, n (%)	87 (92.6)	94	87 (92.6)	94	261 (92.6)	282
Black participants, n (%)	58 (61.7)	94	58 (61.7)	94	180 (68.4)	267
Maximum education, mean (SD)	15.5 (2.4)	94	15.5 (2.4)	94	15.8 (2.5)	282
BMI (kg/m^2^), mean (SD)	46.6 (7.7)	90	34.3 (6.4)	80	33.5 (7.6)	273
Weight (kg), mean (SD)	126.8 (25.2)	89	92.2 (19.3)	80	91.9 (23.1)	273
Cholesterol (mg/dL), mean (SD)	184.5 (36.5)	90	179.0 (37.5)	78	194.7 (41.7)	270
LDL-C (mg/dL), mean (SD)	112.5 (33.3)	91	91.6 (27.2)	78	116.3 (34.6)	268
HDL-C (mg/dL), mean (SD)	49.8 (15.1)	93	69.9 (29.8)	78	58.5 (17.4)	270
Triglycerides (mg/dL), mean (SD)	114.6 (76.3)	91	87.3 (39.9)	78	100 (55.3)	270
Systolic BP (mm Hg), mean (SD)	121.3 (18.1)	94	120.1 (20.1)	81	118.8 (17.4)	273
Diastolic BP (mm Hg), mean (SD)	78.8 (12.0)	94	73 (12.2)	81	74.6 (11.5)	273
BP medication, n (%)	32 (34.0)	94	26 (32.1)	81	90 (33.2)	271
Hypertension, n (%)	42 (44.7)	94	35 (43.2)	81	109 (40.0)	273
Glucose (mg/dL), mean (SD)	102.8 (33.3)	94	89.7 (14.1)	78	98.2 (25.2)	267
Insulin (μU/mL), mean (SD)	21.2 (15.8)	94	9.9 (8.7)	78	12.9 (7.8)	265
HOMA-IR, mean (SD)	5.77 (4.77)	90	2.34 (2.34)	78	3.33 (2.61)	265
Log-hsCRP, mean (SD)	2.04 (0.85)	68	0.83 (0.67)	44	1.32 (0.84)	163
Prevalent diabetes, n (%)	18 (19.2)	94	6 (7.1)	84	34 (12.4)	274
Prevalent MetS, n (%)	47 (50.0)	94	17 (20.1)	81	88 (32.4)	272

Both pre- and postoperative time points (T_0_ and T_1_) are specified for those who underwent a bariatric procedure. Nonsurgical controls were matched at the T_1_ time point.

* T_0_=preoperative CARDIA research visit directly before bariatric procedure (2.3 ± 1.6 years before BarS).

† T_1_=postoperative CARDIA research visit directly following bariatric procedure and the exam visit of matching (2.8 ± 1.7 years after BarS).

BMI indicates body mass index; BP, blood pressure; CARDIA, Coronary Artery Risk Development in Young Adults; HDL-C, high-density lipoprotein cholesterol; LDL-C, low-density lipoprotein cholesterol; MetS, metabolic syndrome; PA, physical activity.

BarS cases and nonsurgical controls were matched at the T_1_ time point, and age, sex, race, education, and BMI at T_1_ were found to be largely balanced between participant groups—indicating successful matching. Mean BMI decreased more than 11 kg/m^2^ in BarS cases between T_0_ and T_1_. However, differences in cardiometabolic risk factors between groups were evident at T_1_. Compared to sex- BMI-matched nonsurgical controls, individuals who underwent a BarS procedure showed lower mean levels of total cholesterol, LDL-C, triglycerides, fasting glucose, and insulin as well as lower prevalences of diabetes and MetS.

**FIGURE 1.**
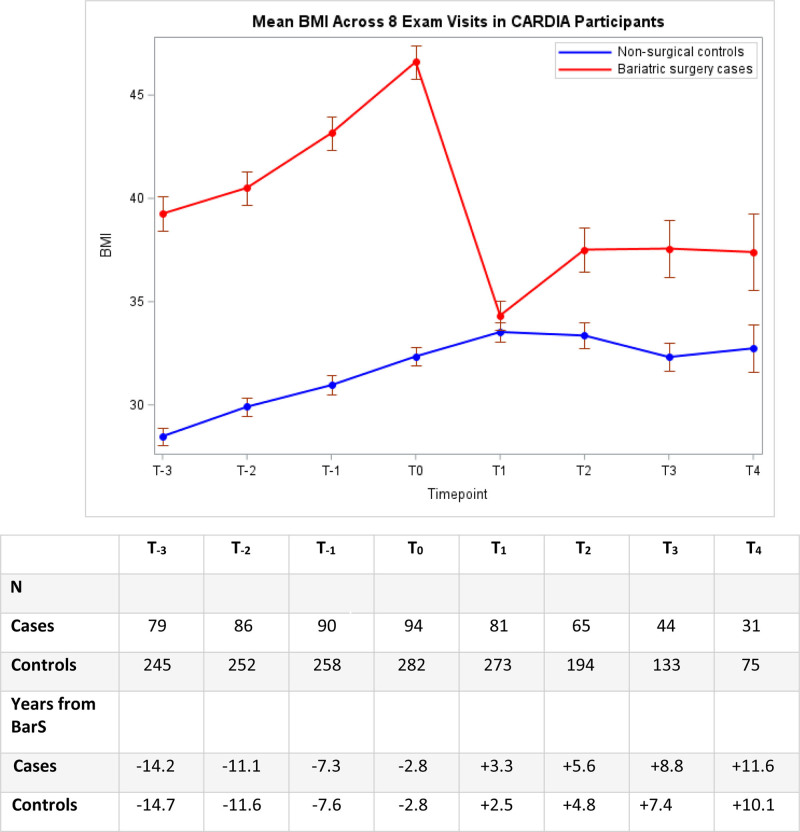
Unadjusted mean BMI (SE) across exam visits in BarS cases and nonsurgical controls.

Trajectories of mean BMI (kg/m^2^) for the BarS and nonsurgical control groups over 8 CARDIA exam visits are shown Figure 1. Compared to nonsurgical controls, individuals in the BarS group showed considerably higher BMIs at the T_−3_ exam visit (+10.8 kg/m^2^) (approximately 15 years before the BarS), and they attained an even higher mean BMI (+14.3 kg/m^2^) by the preoperative time point (T_0_). In the BarS group, mean BMI increased by +7.4 kg/m^2^ between T_−3_ and T_0_ exam visits. By comparison, the trajectory of mean BMI for the nonsurgical controls was less severe (+3.9 kg/m^2^ between T_−3_ and T_0_), and individuals were relatively weight stable by T_1_.

**FIGURE 2.**
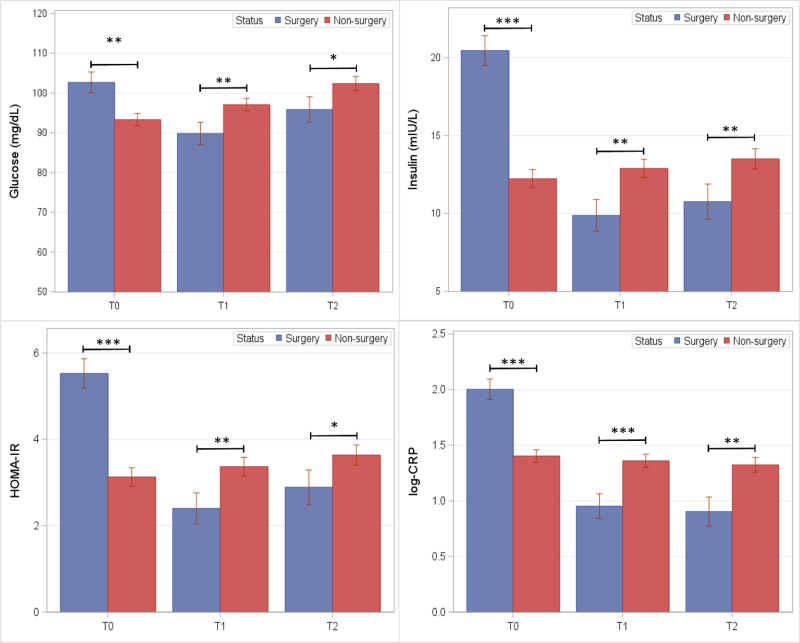
Mean levels (SE) of fasting glucose, insulin, HOMA-IR, and C-reactive protein among BarS and nonsurgical control groups at preoperative (T_0_) and postoperative exam visits (T_1_ and T_2_). *P ≤ 0.05, **P ≤ 0.01, and ***P ≤ 0.001.

**FIGURE 3.**
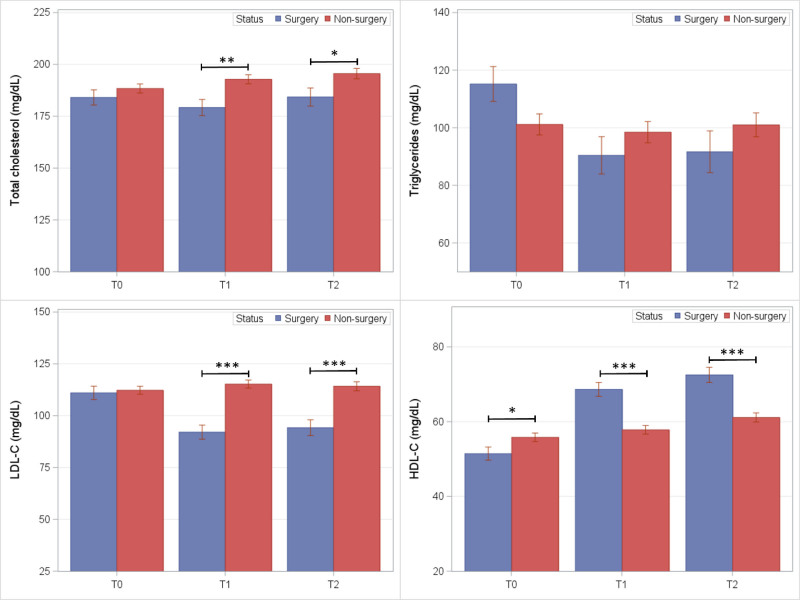
Mean lipid levels (SE) among BarS cases and nonsurgical controls at preoperative (T_0_) and postoperative exam visits (T_1_ and T_2_). *P ≤ 0.05, **P ≤ 0.01, and ***P ≤ 0.001.

**FIGURE 4.**
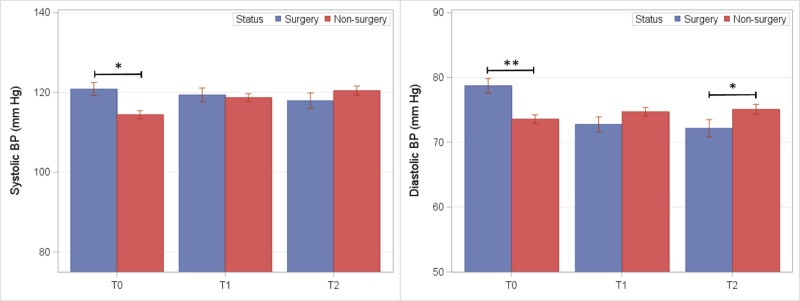
Blood pressure readings among BarS and nonsurgical control groups at preoperative (T_0_) and postoperative exam visits (T_1_ and T_2_). *P ≤ 0.05 and **P ≤ 0.01.

**FIGURE 5.**
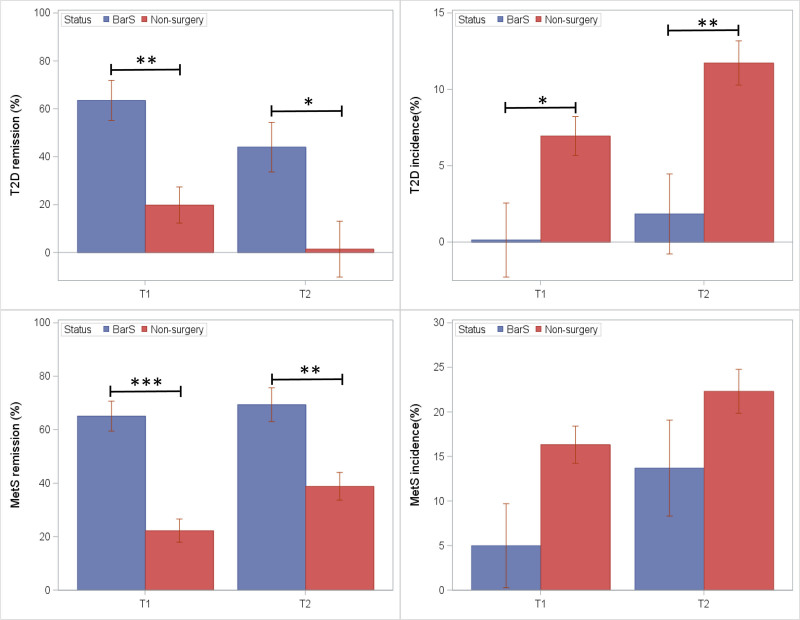
Diabetes and MetS incidence and remission among BarS cases and nonsurgical controls at postoperative exam visits (T_1_) and (T_2_). *P ≤ 0.05, **P ≤ 0.01, and ***P ≤ 0.001.

Mixed model results for cardiometabolic risk factors and adverse metabolic events are shown in Figures 2–5. Fasting levels of glucose and insulin, HOMA-IR, and hsCRP (log-transformed) are shown in Figure 2. At both T_1_ and T_2_ follow-up exams, lower mean levels were observed in the BarS group compared to controls for fasting glucose (T_1_: −7.3 mg/dL; *P* = 0.008; T_2_: −6.5 mg/dL; *P* = 0.03), fasting insulin (T_1_: −3.01 μU/mL; *P* = 0.004; T_2_: −2.75 μU/mL; *P* = 0.01), HOMA-IR (T_1_: −0.97; *P* = 0.004; T_2_: −0.75; *P* = 0.02), and hsCRP (log transformed) (T_1_: −0.41; *P* = 0.001; T_2_: −0.42; *P* = 0.002).

Fasting lipid levels in BarS and nonsurgical control groups are shown in Figure 3. At both follow-up exams, lower mean levels were observed in the BarS group compared to controls for total cholesterol (T_1_: −13.6 mg/dL; *P* = 0.002; T_2_: −11.3 mg/dL; *P* = 0.03) and LDL-C (T_1_: −23.2 mg/dL; *P* < 0.001; T_2_: −20.0 mg/dL; *P* < 0.001). Mean HDL-C was significantly greater in the BarS group at (T_1_: +10.8 mg/dL; *P* < 0.001; T_2_: +11.4 mg/dL; *P* < 0.001). Triglyceride levels were lower in the BarS group, but statistically nonsignificant between groups.

Mean blood pressure in BarS and nonsurgical control groups are shown in Figure 4. Following covariate adjustments, no significant differences between groups were observed at either follow-up exam.

Stratified analyses were performed to examine incidence or remission of diabetes or MetS. For participants in whom disease was absent at T_0_ (Fig. 5; right panels), the BarS group showed blunted disease incidence at T_1_ and T_2_ compared to nonsurgical controls. The estimated incidence of diabetes was lower in BarS cases compared to nonsurgical controls at postoperative T_1_ (0.0% vs 6.9%; *P* = 0.05) and T_2_ (1.8% vs 11.7%; *P* = 0.007). Nominally but nonsignificantly lower MetS incidence was observed in BarS cases compared to nonsurgical controls at postoperative T_1_ (5.0% vs 16.3%; *P* = 0.09) and T_2_ (13.7% vs 22.3%; *P* = 0.23). For participants with prevalent metabolic disease at T_0_ (Fig. 5; left panels), the BarS group showed greater remissions of diabetes at T_1_ (63.5% vs 19.9%; *P* = 0.006) and T_2_ (44.0% vs 1.5%; *P* = 0.03) and remission of MetS at T_1_ (65.1% vs 22.2%; *P* < 0.001) and T_2_ (69.4% vs 38.8%; *P* = 0.005) compared to nonsurgical controls. Among BarS cases, 16 were diabetic at T_0_, and 10 showed remission with medication cessation at the T_1_ follow-up. No individual who underwent BarS developed incident diabetes at the first follow-up exam (T_1_); however, 7 BarS cases developed diabetes by T_2,_ with 5/7 initiating diabetes medications. By contrast, 18 of 254 nonsurgical controls developed diabetes at T1. Of these, 17/18 initiated diabetes medications or were newly detected at T_1._ By T2, 43/163 nonsurgical controls showed prevalent diabetes with 21/43 taking medications. Overall, we found that participants with diabetes who underwent BarS were taking antidiabetic medication at T_0_ and ceased taking medication when their diabetes resolved.

An additional analysis of metabolic outcomes was performed in which failure to improve served as the outcome variable, and this combined incident diabetes or MetS with failure to undergo remission of either diabetes or MetS with adjustment for age, race, sex, and baseline diabetes and MetS status. Shown in Figure S14, https://links.lww.com/AOSO/A533, the BarS group showed significantly lower proportions of individuals who failed to improve compared to the nonsurgical controls at T_1_ (12.1% vs 34.6%; *P* < 0.001) and T_2_ (14.1% vs 35.1%; *P* < 0.001).

To ensure fidelity with previous studies, a secondary analysis was performed that matched surgical cases with nonsurgical controls at the preoperative time point (T_0_). Characteristics of BarS surgery cases and nonsurgical controls matched at T_0_ are shown in Table S1, https://links.lww.com/AOSO/A533. For cardiometabolic outcomes, glucose, insulin, HOMA-IR, hsCRP (log-transformed), cholesterol, and blood pressure across the pre- and postoperative time points are shown in Figures S15–S17, https://links.lww.com/AOSO/A533. No significant differences were observed between groups at the preoperative time point, consistent with a successful match. Compared to nonsurgical controls, BarS cases showed significantly lower levels of glucose, insulin, HOMA-IR, hsCRP (log-transformed), LDL-C, HDL-C, triglycerides, and diastolic blood pressure at postoperative time points T_1_ and T_2_. Similarly, metabolic outcomes as determined by failure to improve are shown in Figure S18, https://links.lww.com/AOSO/A533. Diabetes and MetS outcomes were not statistically different at the preoperative time point (T_0_), but both were lower among BarS cases at both postoperative time points T_1_ and T_2_.

## DISCUSSION

This study examined longitudinal outcomes among individuals who underwent a bariatric procedure achieve long-term cardiometabolic health that is consistent with the lower weight status attained in the years following surgery or, in contrast, whether lasting consequences of severe obesity from the years before surgery are evident in the years following surgery. Contrary to our hypothesis, individuals who underwent BarS were significantly less likely to develop diabetes compared to nonsurgical controls while showing significant remissions of diabetes and MetS over the approximate 7.5 years following their procedures. The BarS group showed a durable and clinically significant increase in HDL-C and lower blood glucose, insulin, total cholesterol, LDL-C, HOMA-IR, and hsCRP compared to nonsurgical controls who were BMI-matched at a postoperative time point. These findings suggest that the long-term health consequences of severe obesity are reversed with surgical intervention, and patients can achieve aspects of cardiometabolic health compared to individuals who never experienced years of severe obesity.

### Mechanisms of Cardiometabolic Health Improvements Following BarS

Previous studies in patients and experimental models have shown that bariatric procedures promote cardiometabolic health through weight-dependent and -independent mechanisms. Changes in bile acid metabolism and composition,^23^ gut peptide secretions,^24^ shifts in microbiota populations,^25^ adipose tissue health and function,^26,27^ lower inflammation,^28^ or some combination of these and other phenomena appear to improve insulin sensitivity and metabolic health following BarS but before weight loss. While we cannot evaluate whether these changes persist in the years following BarS in this sample, they are consistent with the significantly lower mean levels of glucose, insulin, and HOMA-IR among BarS cases observed here. In addition, the greater mean HDL-C levels among Bars cases may be due to well-described increases in adiponectin and cholesterol efflux following surgery^29–32^ as well as the influence of adiponectin on HDL function and its primary protein component, apolipoprotein A1.^33–35^ Whether these or other mechanisms contributed to the lasting associated improvements in cardiometabolic risk profiles in the BarS group compared to postoperatively matched controls is unclear but warrants further investigation.

### Implications for Obesity Interventions and Further Research

The present findings have a number of implications. Foremost, our results emphasize the importance of surgical intervention in those with severe obesity with or without compromised metabolic health. On average, those who underwent BarS attained superior cardiometabolic health profiles and blunted incidence or greater remissions of metabolic disease compared to individuals who never experienced years of severe obesity over the follow-up. Further, BarS cases returned to a cardiometabolic health phenotype similar to themselves 17 years earlier (comparing T_−3_ and T_1_ exam visits, Figures S1–S12, https://links.lww.com/AOSO/A533). These findings extend those of previous studies by showing that BarS appears to reverse the associated consequences of severe obesity and instill further benefits when compared to the nonsurgical postoperatively matched individuals.

Consistent with other studies of BarS, we observed considerable heterogeneity in cardiometabolic outcomes at follow-up exams. The underlying reasons for this heterogeneity are multifactorial and overlapping, but variability in patient histories, comorbidities, genetic liabilities, psychosocial factors, appetite and satiety signaling, and behaviors likely contribute. For example, we observed a highly variable increase in self-reported physical activity in the CARDIA exam following surgery, ~2.5 years postop, which abated 4.8 to 7.4 years following surgery (Figure S13, https://links.lww.com/AOSO/A533). This phenomenon is consistent with a meta-analysis of 26 studies showing an increase in physical activity directly following BarS with high heterogeneity (*I*^2^ = 83%)^36^ but that ultimately returns to preoperative levels for most patients within 3 to 7 years of their procedures.^37^ The variability of physical activity and other factors likely contributes to overall health outcomes among BarS patients. And while these have been examined individually or in select combinations,^36–41^ more comprehensive approaches are warranted due to the complexity of the underlying pathophysiology, likely interactions between patient characteristics (eg, psychosocial and physical activity), the imprecision in some self-reported measurements, and unmeasured confounders. We posit that collective examination of behavioral, psychosocial, and physiological factors that promote durable metabolic health and constrain weight regain will advance the precision medicine of BarS. Moreover, such an approach will allow for individualized patient care plans including dietary recommendations, adjunct pharmacological therapies, and omics-based targets for patient monitoring.

### Strengths and Limitations

There were several strengths to our analysis and cohort-based approach. First, in contrast to the many retrospective studies that rely on patient registries and electronic health records, the CARDIA cohort is a well-phenotyped community-based cohort, which allowed for adjustment for maximum education attainment—an important social determinant of health. In addition, CARDIA exam visits allowed for the interrogation of cardiometabolic health profiles and the presence of metabolic disease in study participants at regular intervals.

Study limitations must also be acknowledged. First, a total of 94 CARDIA participants were available for analysis at the preoperative exam visit (T_0_), and fewer individuals were available at preceding or subsequent exam visits. This limited our ability to test longitudinal differences between the BarS cases and nonsurgical controls and potential modifying influences of socioeconomic status or behavior variables. Second, the bariatric procedure exposure was a composite of vertical sleeve gastrectomy, gastric bypass, gastric banding, or self-reported bariatric or weight loss procedure that was otherwise unknown. We did not have the statistical power to examine procedures separately, and differential associations based on the procedure type are likely. Third, the bariatric procedures occurred throughout the CARDIA study, and most exam visits were approximately 5 years apart. This represents a source of imprecision, since we could not fully capture peak presurgical weights or subsequent anthropometric and metabolic improvements. Fourth, weight regain following BarS has been shown to coincide with poorer metabolic health including increases in glucose, insulin, or glycosylated hemoglobin.^42–44^ In this sample, we did not have the statistical power to evaluate the differences in cardiometabolic health based on weight regain over the follow-up period. Further studies of the cardiometabolic outcomes associated with weight regain, its heterogeneity among BarS patients, and mitigation strategies are warranted. Finally, and in terms of cardiovascular outcomes, CARDIA does not have imaging such as carotid ultrasound or computed tomography-derived coronary artery calcium across multiple visits, so we were unable to evaluate the potential for irreversible increases in atherosclerotic burden of severe obesity among individuals who underwent BarS.

## CONCLUSIONS

We found no evidence that years of obesity or severe obesity imposed irreversible cardiometabolic health consequences in individuals following their BarS procedures for these select cardiometabolic outcomes. Further studies are warranted to explore other outcomes, like atherosclerotic burden as well as the observed heterogeneity in improvements following BarS and the extent to which weight regain may explain this heterogeneity.

## ACKNOWLEDGMENTS

We thank the participants, staff, and investigators of the CARDIA study for their dedication and highly valued contributions. This manuscript was reviewed for scientific content by the CARDIA P&P committee before submission to the journal.

## Supplementary Material
