# Supplementary material for: Cardiometabolic Health and Bariatric Surgery: A 25-Year Longitudinal Cohort Study in CARDIA Participants
Source: Ann Surg Open. 2025 Sep 4;6(3):e609. doi: 10.1097/AS9.0000000000000609 (PMC12453380; doi:10.1097/AS9.0000000000000609)
Supplement: Supplementary file 1 [file as9-6-e609-s001.pdf]

Supplementary Material

Supplementary eFigures 1-12. Characteristics of bariatric surgery patients and non-surgical controls (sex- BMI-matched at T_1) across approximately 25 years at 8 CARDIA exam visits.

eFigure 1

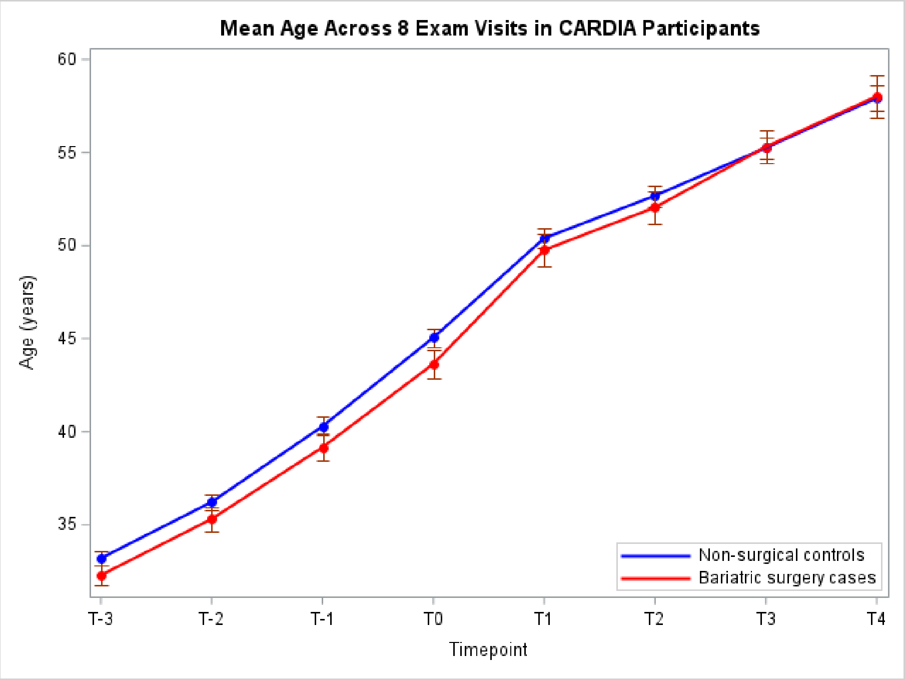

eFigure 2

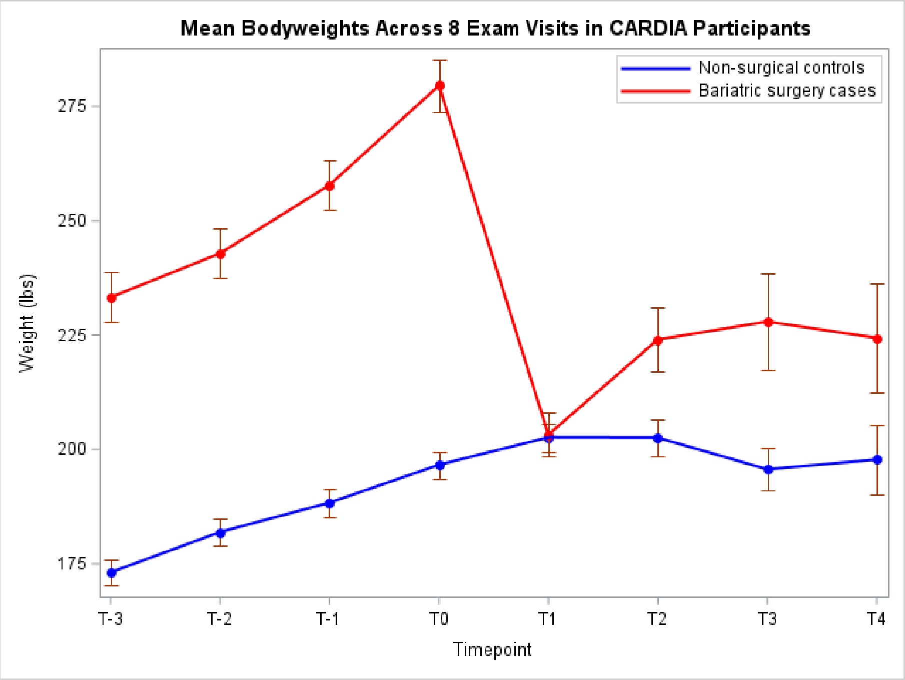

eFigure 3

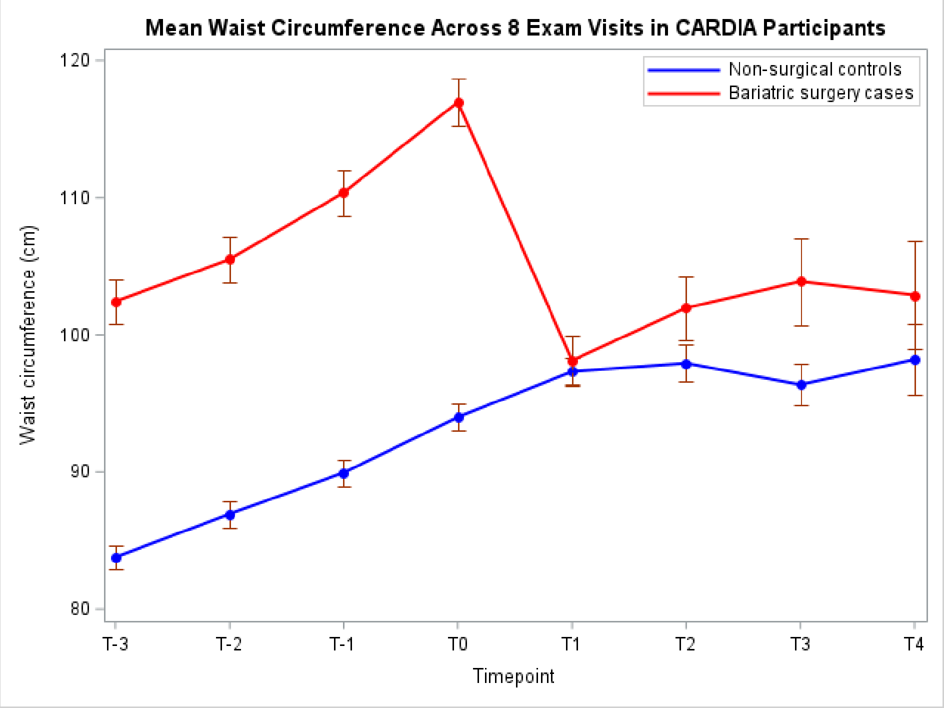

eFigure 4

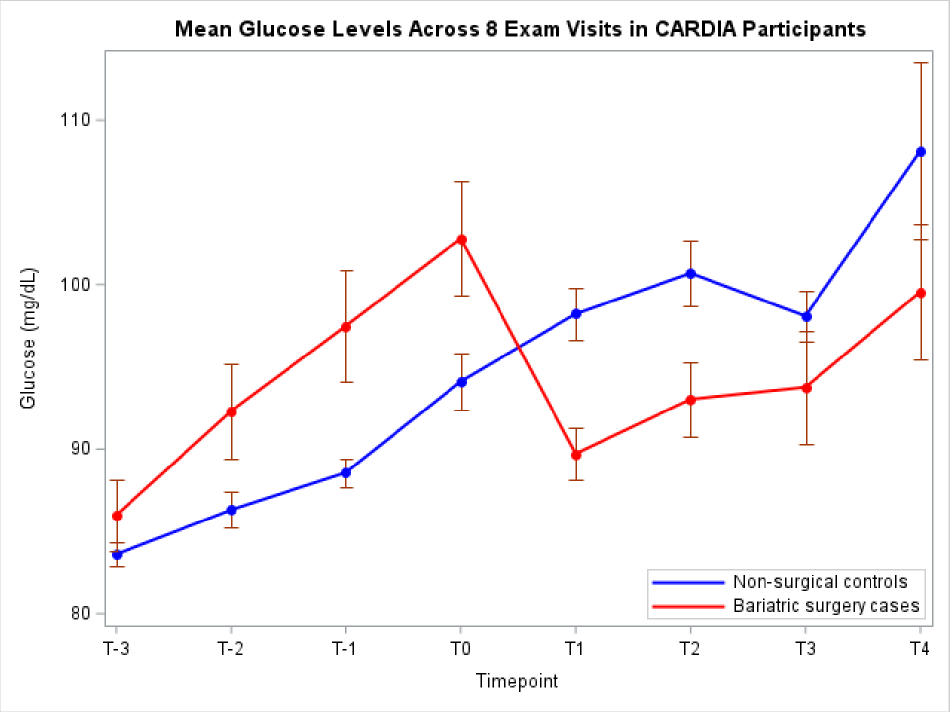

eFigure 5

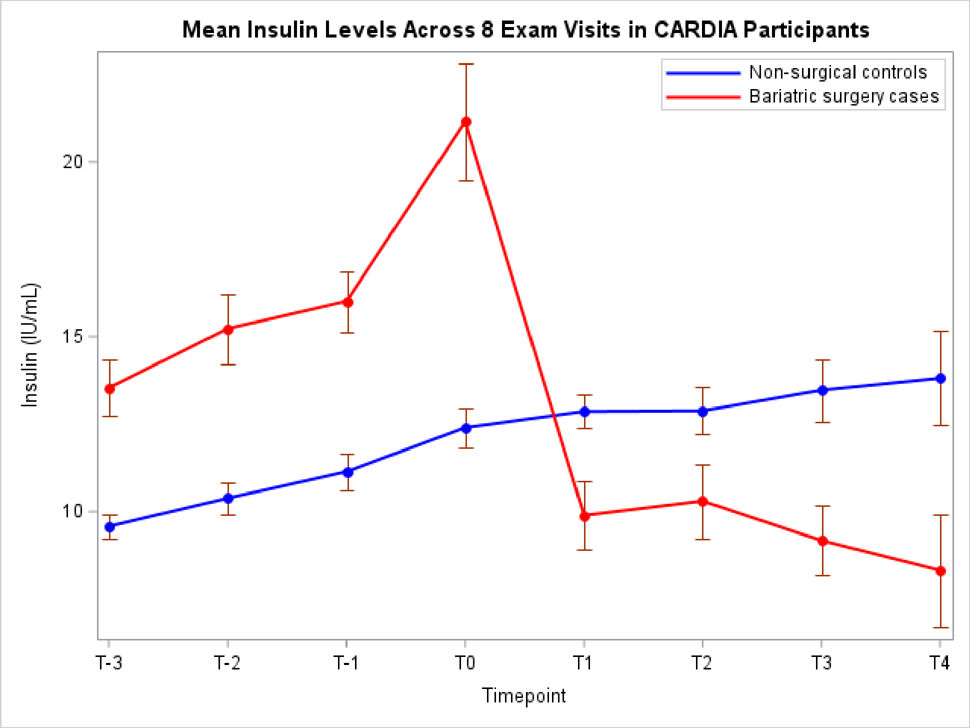

eFigure 6

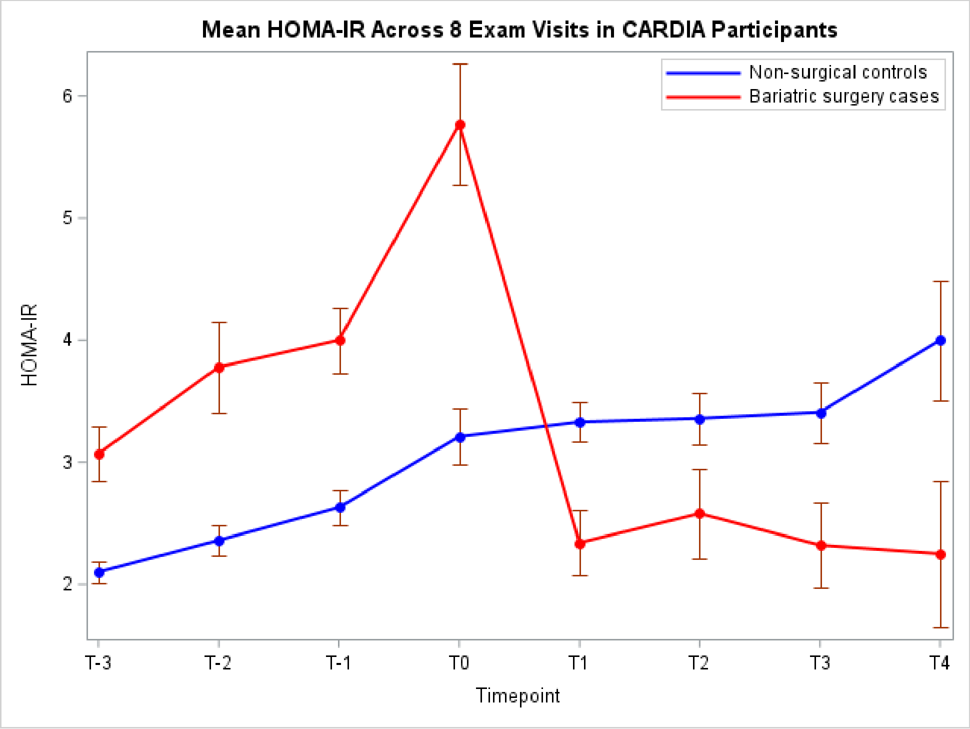

eFigure 7

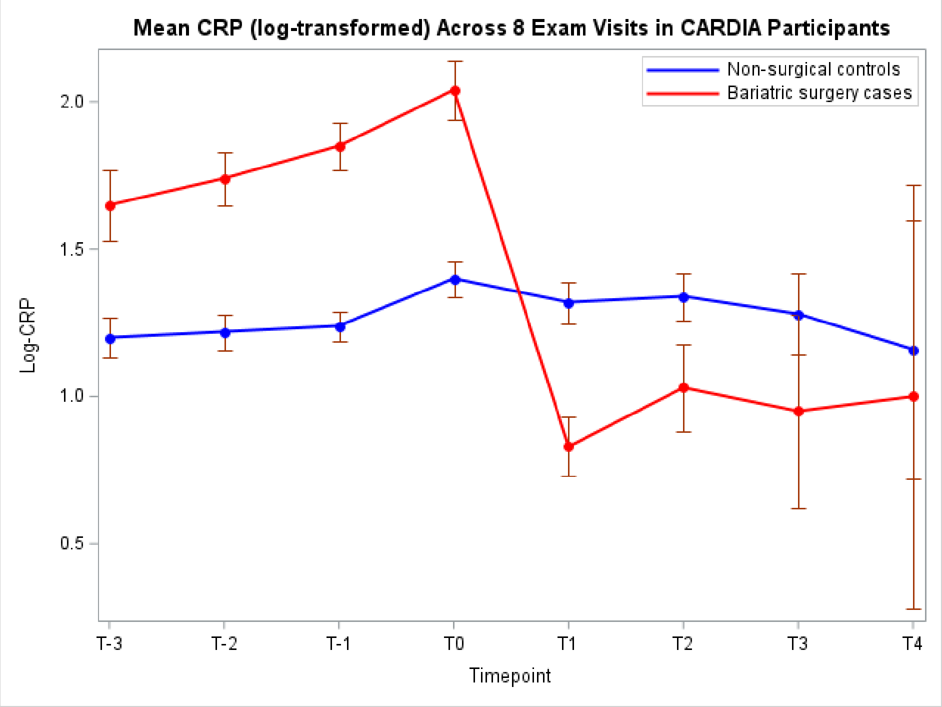

eFigure 8

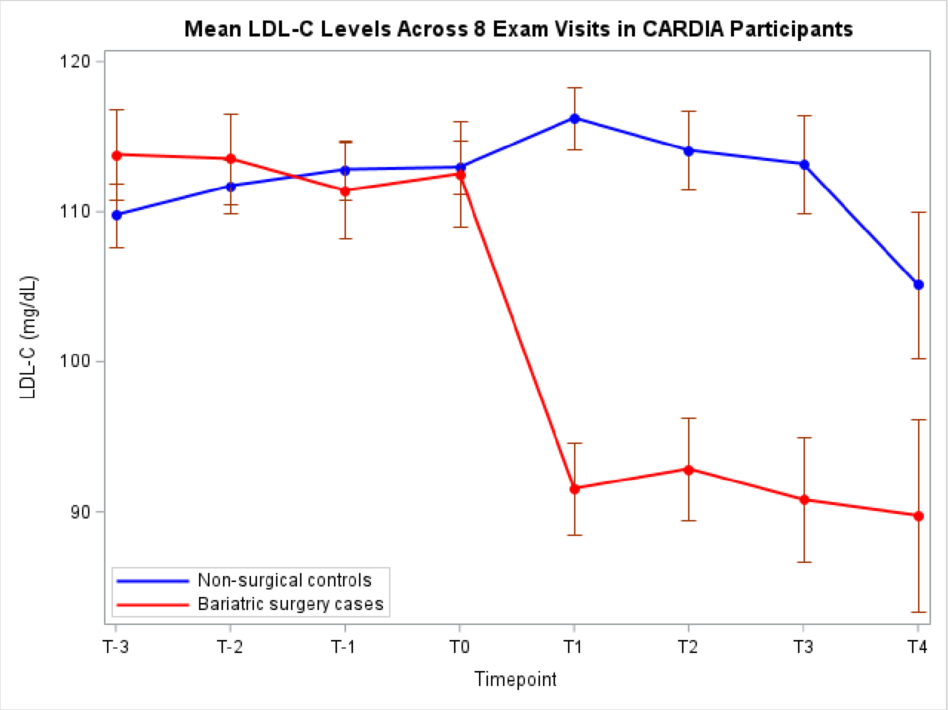

eFigure 9

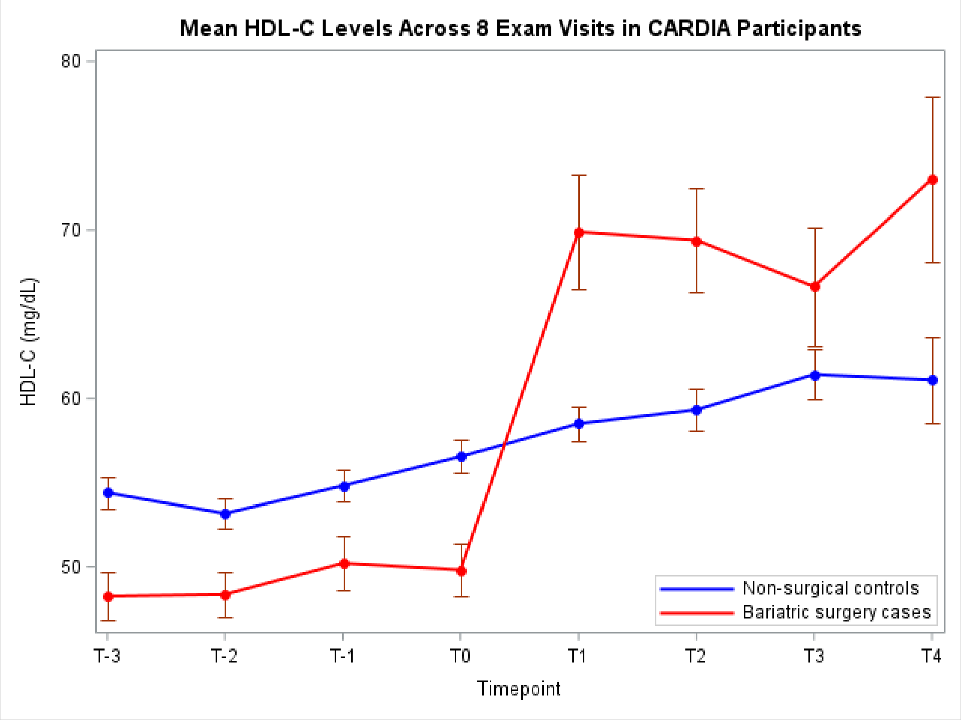

eFigure 10

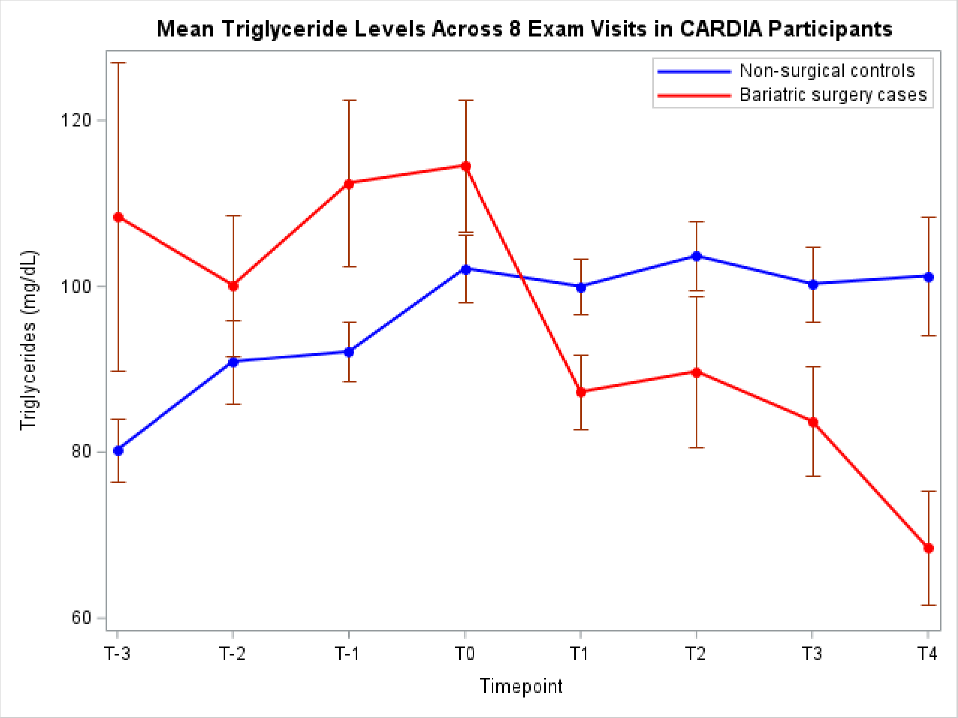

eFigure 11

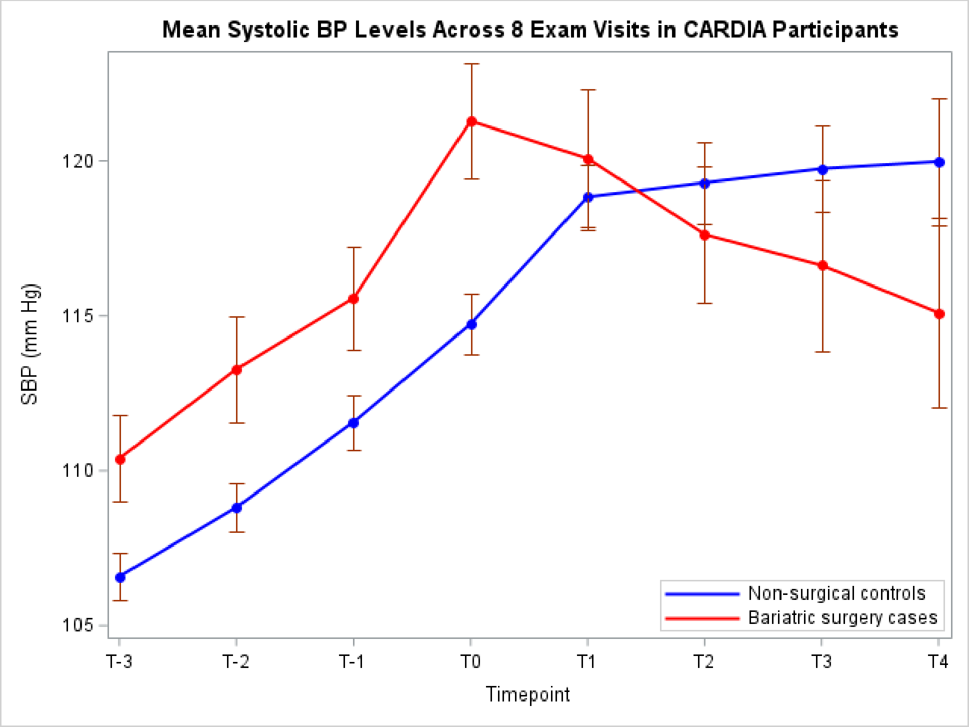

eFigure 12

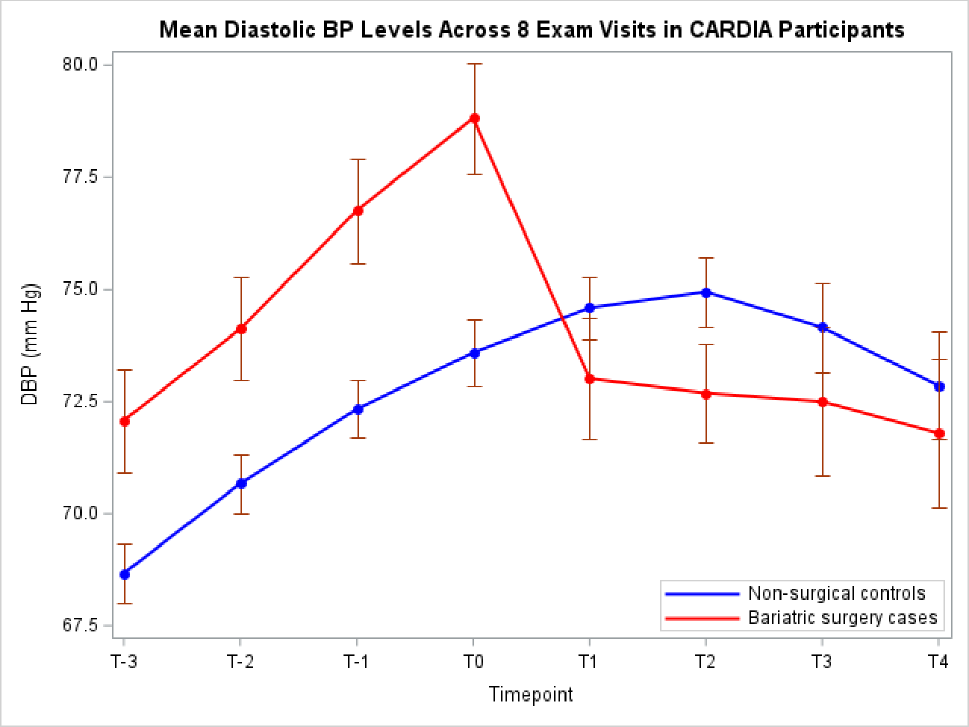

eFigure 13

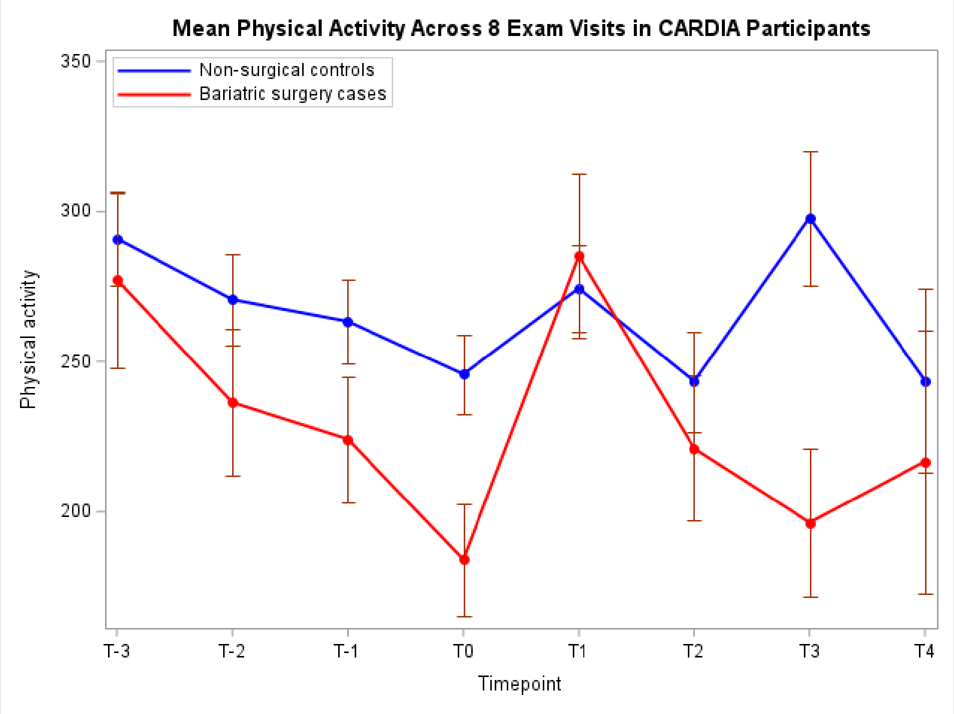
Mean Physical Activity Across 8 Exam Visits in CARDIA Participants
Non-surgical controls
Bariatric surgery cases
Physical activity
350
300
250
200
T-3
T-2
T-1
T0
T1
T2
T3
T4
Timepoint

eFigure 14. Failure to improve* (%) at postoperative time points among BarS and non-surgical control groups at postoperative exam visits (T1 and T2).

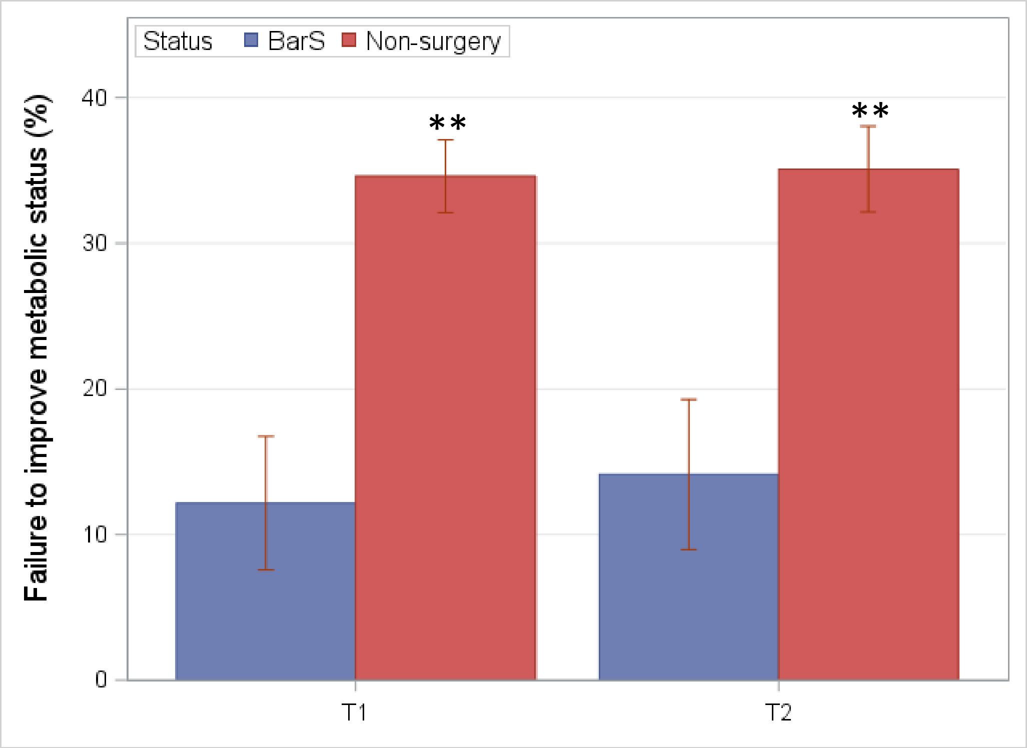

*Failure to improve combined incident disease from the preoperative time point with failure to undergo remission of either diabetes or MetS

**Significantly different than BarS at corresponding time point (p<0.001)

eTable 1. Demographic, lifestyle, and clinical characteristics of CARDIA participants who underwent a bariatric procedure and BMI-matched controls at preoperative time point (T_0).

Characteristic	Surgery cases (T_0)	N	Non-surgical controls (T_0)	N
Age (yr), mean (SD)	43.6±7.3	94	44.8±7.5	267
Age at surgery (yr), mean (SD)	46.5±8	94	47.6±8.2	267
Time between T_0 and surgery, mean (SD)	2.3±1.6	94	--	--
Time from surgery to T_1 (yr), mean (SD)	2.8±1.7	94	2.8±1.7	267
Female, n (%)	87 (92.6)	94	249 (94.7)	267
Black participants, n (%)	58 (61.7)	94	180 (68.4)	267
Maximum education, mean (SD)	15.5±2.4	94	15.3±2.5	267
Total PA (exercise units), mean (SD)	184±180	91	196±205	258
BMI (kg/m^2), mean (SD)	46.6±7.7	94	44.0±6.6	267
Weight (lbs), mean (SD)	279.5±55.5	94	264.3±46.9	262
Cholesterol (mg/dL), mean (SD)	184.5±36.5	90	185.4±33.6	257
LDL-C (mg/dL), mean (SD)	112.5±33.3	89	113.3±29.8	254
HDL-C (mg/dL), mean (SD)	49.8±15.1	90	50.5±13.3	257
Ln triglycerides, mean (SD)	4.6±0.5	91	4.6±0.5	260
Cholesterol medication, n (%)	13 (14)	93	21 (8.1)	260
SBP (mm Hg), mean (SD)	121.3±18.1	94	119.4±14.9	267
DBP (mm Hg), mean (SD)	78.8±12	94	78.3±10.5	267
BP medication, n (%)	32 (34.0)	94	94 (35.7)	267
Hypertension, n (%)	42 (44.7)	94	123 (46.8)	267
Glucose (mg/dL), mean (SD)	102.8±33.3	91	100.4±23.6	260
Insulin (µU/mL), mean (SD)	21.2±15.8	90	18.5±10	255
Prevalent diabetes, n (%)	18 (19.2)	94	45 (17.1)	267
Prevalent MetS, n (%)	47 (50.0)	94	130(49.6)	262

Abbreviations: PA physical activity; BMI body mass index; LDL-C low density lipoprotein cholesterol; HDL-C high density lipoprotein cholesterol; Ln natural logarithm; SBP systolic blood pressure; DBP systolic blood pressure; MetS metabolic syndrome.

eFigure 15. Mean levels (SE) of fasting glucose, insulin, HOMA-IR, and C-reactive protein among at preoperative (T_0) and postoperative exam visits (T_1 and T_2) *. BarS cases and non-surgical controls were matched at the preoperative time point (T_0).

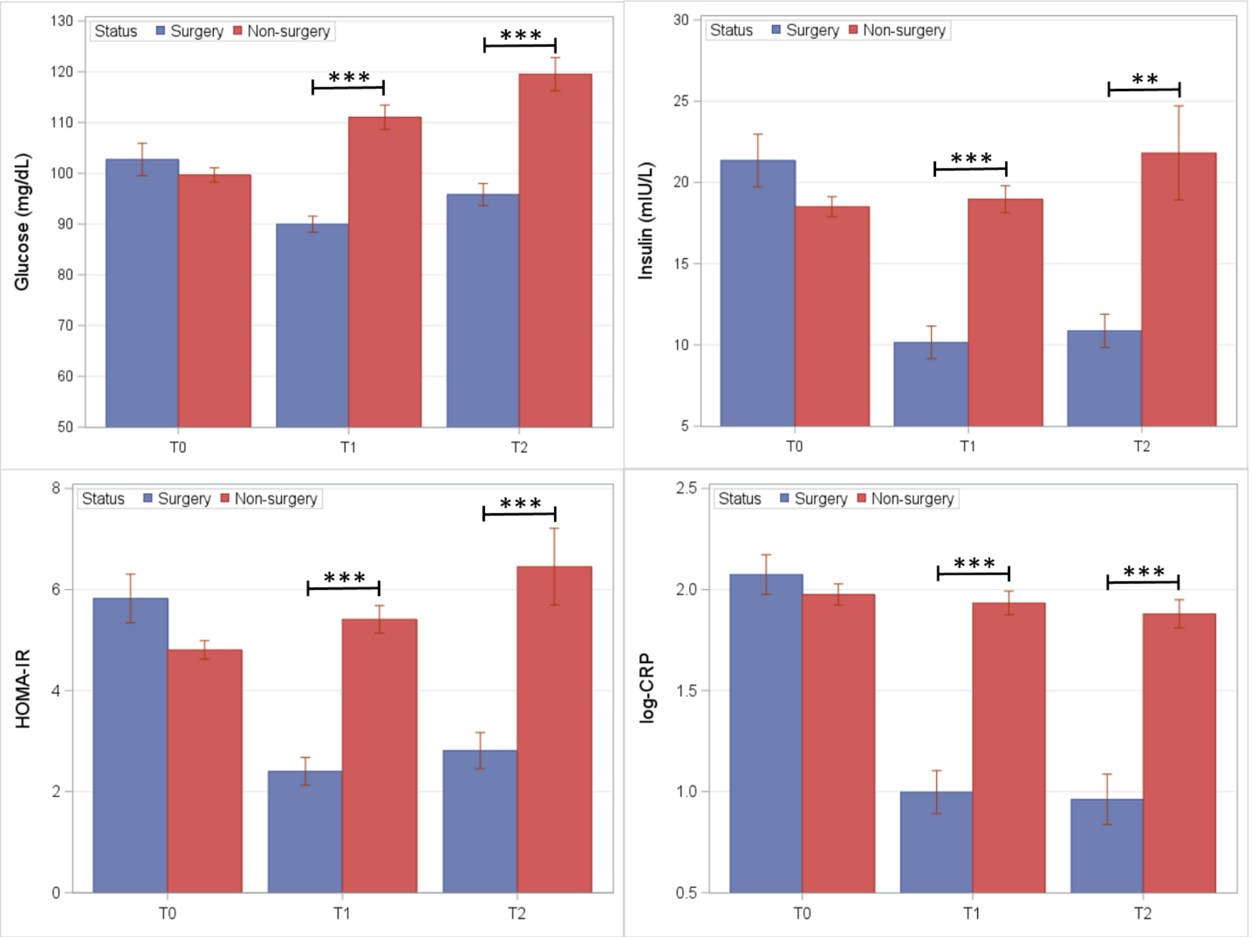

*Significance tests are for net difference from T0

***p*<0.001

****p*<0.0001

eFigure 16. Mean lipid levels (SE) among BarS cases and non-surgical controls at preoperative (T0) and postoperative exam visits (T1 and T2) *. BarS cases and non-surgical controls were matched at the preoperative time point (T_0).

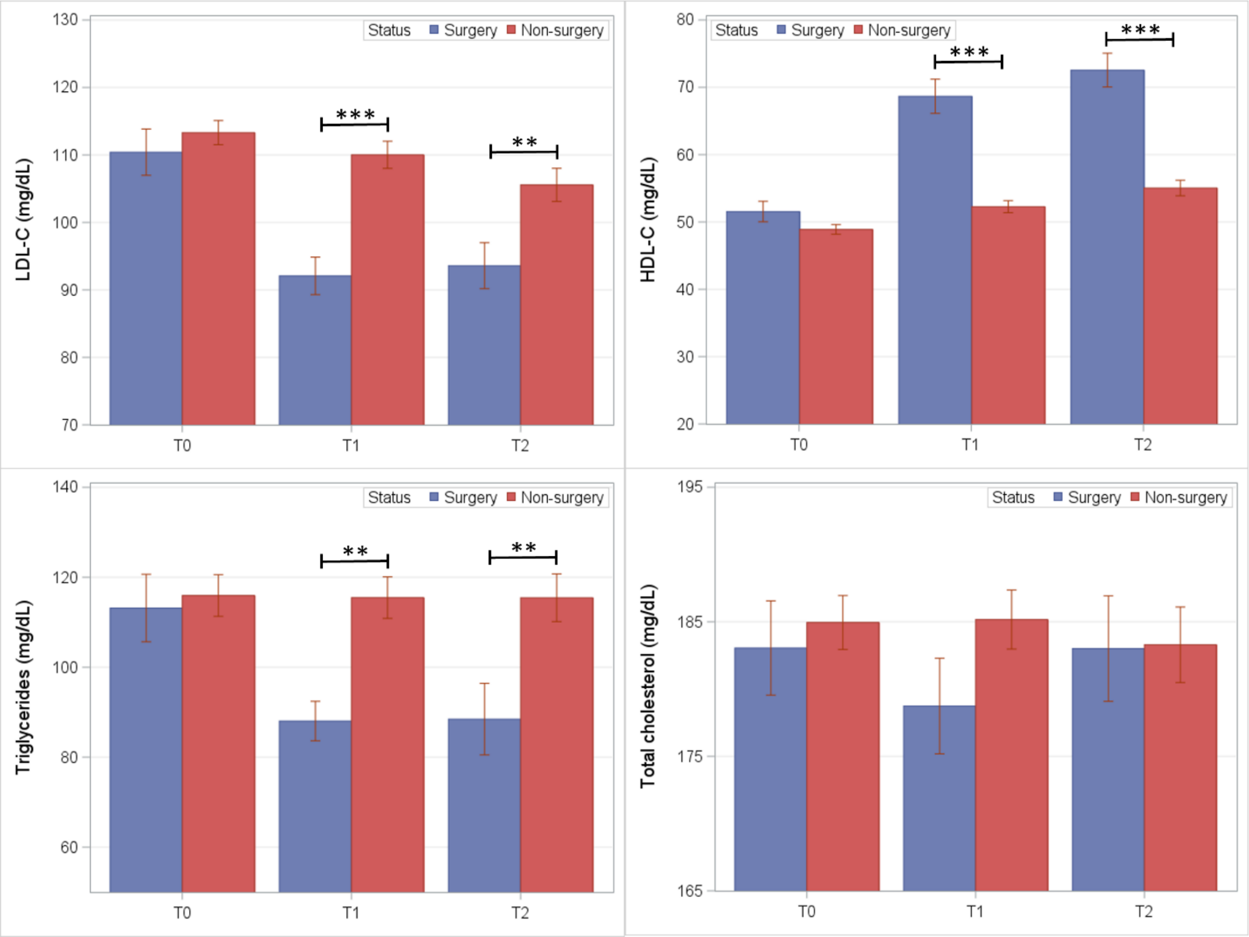

*Significance tests are for net difference from T0

***p*<0.001

****p*<0.0001

eFigure 17. Blood pressure readings among BarS and non-surgical control groups at preoperative (T_0) and postoperative exam visits (T_1 and T_2) *. BarS cases and non-surgical controls were matched at the preoperative time point (T_0).

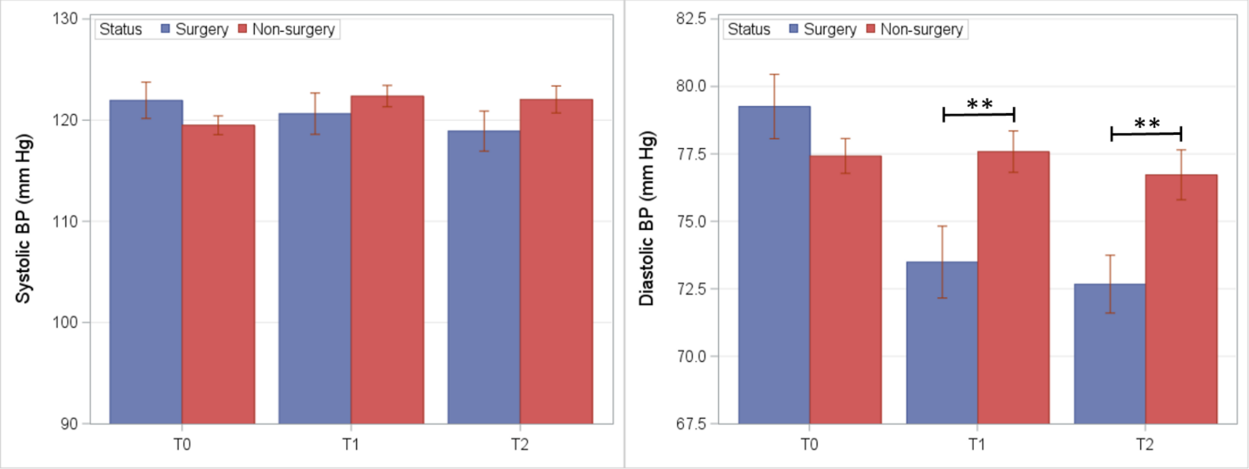

*Significance tests are for net difference from T0

***p<0.001

eFigure 18. Failure to improve* (%) at postoperative time points among BarS and non-surgical control groups at postoperative exam visits (T1 and T2) **. BarS cases and non-surgical controls were matched at the preoperative time point (T_0).

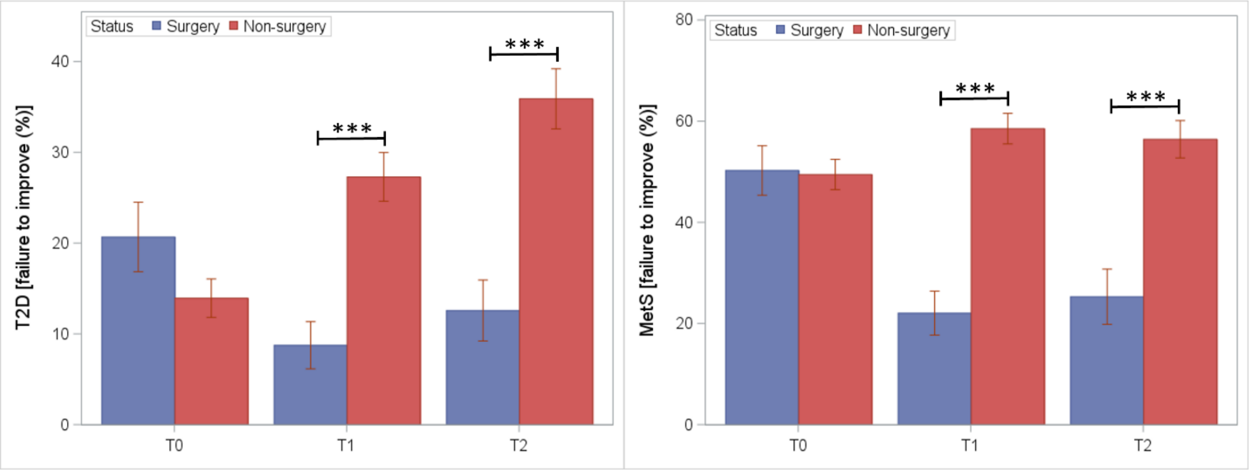

*Failure to improve is defined as prevalent disease at T_0 and either incident disease or failure to undergo remission at T_1 or T_2.

**Significance tests are for net difference from T0

***p<0.0001
